# Diffuse Large B-Cell Lymphoma: Dramatic Evolution From Unilateral to Bilateral Leg Edema

**DOI:** 10.7759/cureus.73659

**Published:** 2024-11-14

**Authors:** Uta Shimada, Yuichiro Mine, Yuichi Takahashi, Gautam A Deshpande, Toshio Naito

**Affiliations:** 1 Clinical Training Center, Juntendo University Hospital, Tokyo, JPN; 2 General Medicine, Juntendo University Faculty of Medicine, Tokyo, JPN

**Keywords:** diffuse large b cell lymphoma (dlbcl), fdg pet/ct scan, leg edema, malignant lymphoma, venous drainage issues

## Abstract

Edema can usually be classified as either systemic (bilateral) or localized (unilateral). Lymphoma-associated leg edema is generally bilateral; there have been several reports of patients presenting with unilateral edema due to unilateral vascular compression.

We describe a case of diffuse large B-cell lymphoma (DLBCL) initially presenting with unilateral edema, which rapidly developed into bilateral edema over two weeks due to vascular compression.

## Introduction

Leg edema is a common symptom in primary care settings, and the time course of the onset is crucial when considering differential diagnoses. Typically, the cutoff for distinguishing between acute and chronic edema is considered to be 72 hours [1,2,3].

The frequency of bilateral edema is most commonly attributed to venous insufficiency in patients over the age of 50. On the other hand, the most likely cause of lower leg edema in women in their 20s to 30s is idiopathic edema, commonly associated with the menstrual cycle and known as cyclic edema [4,5].

Most unilateral edema is usually caused by local factors such as deep vein thrombosis, venous insufficiency, or lymphedema. Specifically, lymphedema can be classified into primary and secondary types, with most cases being secondary lymphedema. Common causes of secondary lymphedema include surgery involving the lymphatic system, radiation therapy, malignancies, and infections caused by filariasis or bacteria. It has also been reported that malignant lymphoma can lead to secondary lymphedema.

Although lymphoma-associated leg edema is generally bilateral [6], there have been several reports of patients presenting with unilateral edema due to unilateral vascular compression [7]. All of these conditions represent chronic edema. However, a few reports discuss the progression of edema from unilateral to bilateral and its time course. This report describes a case of diffuse large B-cell lymphoma (DLBCL), which initially presented with unilateral edema, which rapidly progressed to bilateral edema within two days of initial presentation, with changes and findings of vascular compression by the tumor captured in a two-week follow-up.

## Case presentation

A 68-year-old Japanese man with a history of descending colon cancer resected eight years ago and otherwise healthy presented to our outpatient clinic with right leg edema. One month prior, the patient noticed unprovoked edema in his right leg and scrotum. The condition worsened, with a weight gain of 1 kg since the initial onset, making it difficult to put on shoes at the time of the visit. The patient denied any history of fever, drenching night sweats, or other constitutional symptoms.

**Figure 1:**
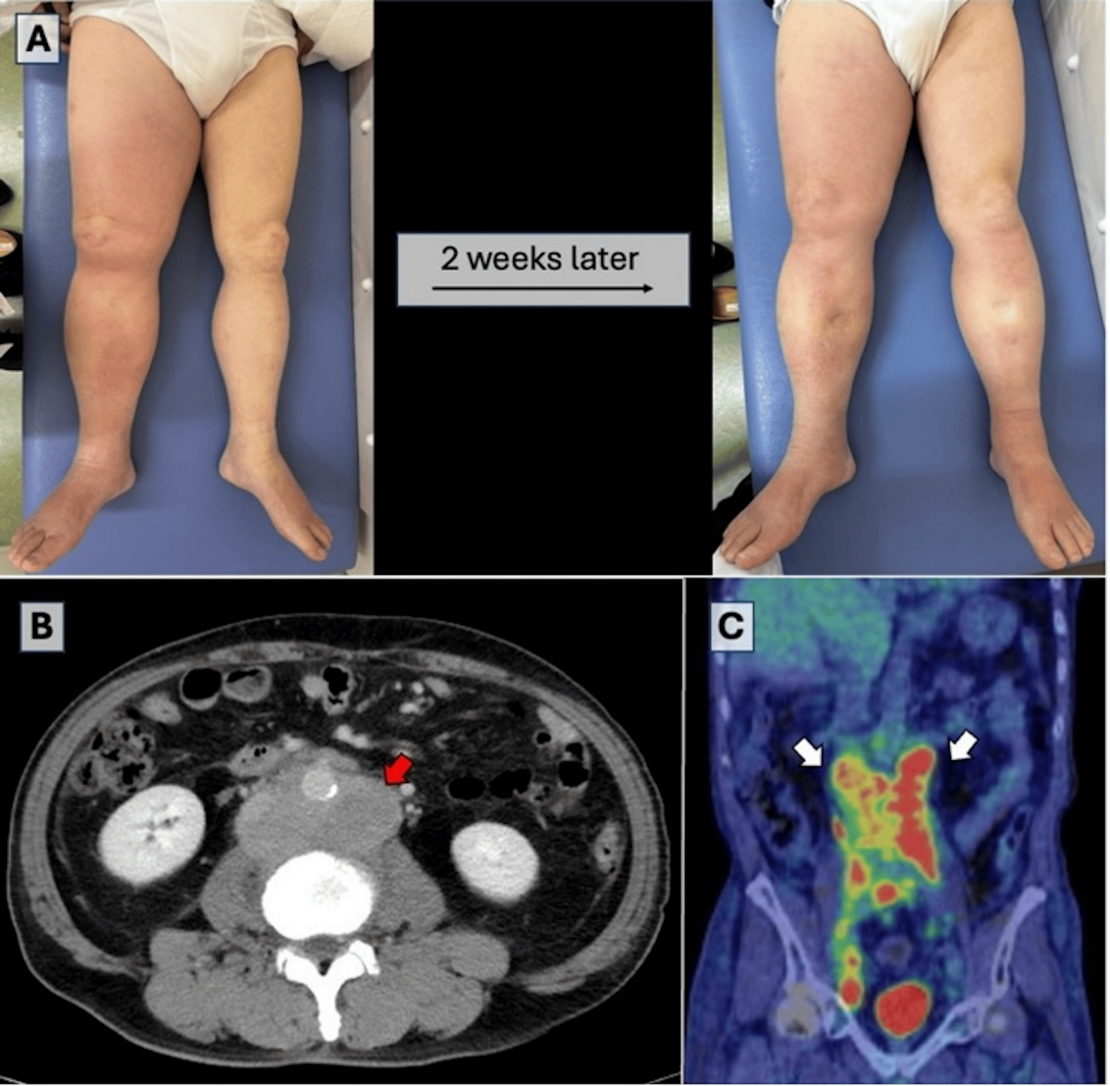
Clinical course of leg edema, abdominal/pelvic CT, and FDG-PET/CT A: leg edema progresses from unilateral to bilateral over two weeks; B: abdominal/pelvic contrast-enhanced CT in the axial view shows enlarged lymph nodes around the paraaortic lesion (red arrow); C: FDG-PET/CT in the coronal view shows increased uptake in multiple enlarged lymph nodes (white arrows). FDG: fludeoxyglucose-18; PET/CT: positron emission tomography/computed tomography

Vital signs were within normal limits. Physical examination revealed 2+ pitting edema of the right leg extending from the inguinal area to the toes, with mild erythema and calf tenderness without warmth or numbness (Figure 1A). No remarkable palpable lymphadenopathy was noted anywhere. A venous Doppler ultrasound did not detect any signs of deep venous thrombosis (DVT) in either leg. Laboratory evaluation revealed elevated ferritin levels of 585 ng/mL and a soluble interleukin-2 receptor level of 3,940 U/mL. Enhanced abdominal and pelvic computed tomography (CT) revealed bulky lymphadenopathy along the abdominal aorta and bilateral iliac arteries compressing the right iliac vein (Figure 1B). Fluorodeoxyglucose-positron emission tomography/computed tomography (FDG-PET/CT) showed multiple enlarged lymph nodes with abnormal FDG uptake in the left axillary, mediastinal, retrocrural, bilateral para-aortic, iliac, and mesenteric areas (Figure 1B, 1C).

**Figure 2:**
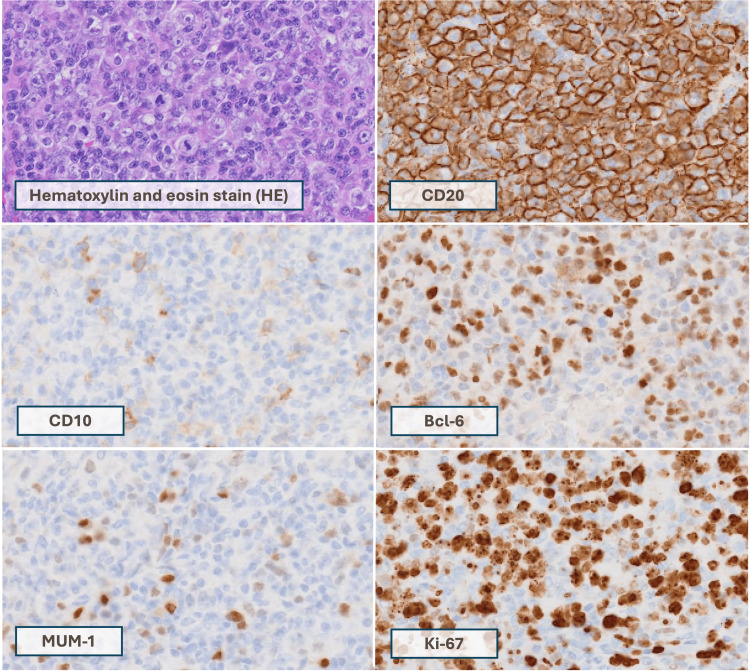
Pathological images of this case Atypical lymphocytes with medium to large nuclei are diffusely proliferating (HE). On immunostaining, they are CD20 positive, CD10 negative, Bcl-6 partially positive, MUM-1 negative, and Ki-67 positive (>90%).

During the next follow-up for two weeks, the patient reported worsening edema on the opposite side within two days of the last visit and was unable to wear shoes. The patient presented with bilateral 4+ pitting edema (Figure 1A). A left axillary lymph node biopsy confirmed high-grade diffuse large B-cell lymphoma (DLBCL) with germinal center B-cell type (Figure 2), and the patient subsequently began combination therapy with rituximab, cyclophosphamide, doxorubicin hydrochloride, vincristine sulfate, and prednisone (R-CHOP).

## Discussion

Leg edema is broadly classified based on whether it is bilateral or unilateral and whether its onset is acute or chronic (Table 1) [8,9,10]. In cases of acute unilateral edema, identifying DVT is essential. In addition to edema, DVT patients may present with calf tenderness, pain or hardness along the course of the veins, unilateral warmth, and erythema. However, Homan's sign (pain in the calf upon dorsiflexion of the foot) is reported to have a sensitivity of 8 to 56% and a specificity of less than 50%. Moreover, up to 30% of patients who test positive for DVT with the Homan's sign do not have DVT, so Homan’s sign is unreliable as a sign of DVT [11]. Therefore, it is crucial to consider the risk of DVT by calculating factors such as the Wells score or modified Wells score and the results of coagulation system evaluation and Doppler ultrasound findings in differentiating DVT. For other conditions like muscle injury or cellulitis, the differential diagnoses include venous insufficiency, lymphatic obstruction, or impaired fluid drainage. Thus, localized diseases affecting capillary fluid movement must be considered. In bilateral edema, the differential diagnosis includes acute or chronic processes such as cardiac, renal, hepatic, endocrine, or pharmaceutical-related conditions, and other systemic diseases affecting water or sodium balance [8]. 

**Table 1:** Differential diagnosis of leg edema DVT: deep venous thrombosis Table credits: Uta Shimada

	Unilateral	Bilateral
Acute	- Baker's cyst rupture	- Acute worsening of chronic causes
	- Cellulitis	- Heart failure
	- DVT	- Medications
	- Knee abnormality	- Nephrotic syndrome
	- Leg swelling in a paralyzed limb	- Urticaria
	- Lymphangitis or lymph obstruction	- Venous thrombosis
	- Muscle strain, tear, or twisting injury to the leg	
	- Venous insufficiency	
Chronic	- Baker's cyst	- Cyclic edema
	- Chronic venous disease	- Dependent edema
	- Complex regional pain syndrome	- Heart failure
	- Lymphedema	- Idiopathic edema
	- Paralysis	- Liver disease (e.g. early cirrhosis)
	- Pelvic neoplasm compromising venous return	- Lymphedema
	- Venous valve insufficiency	- Malnutrition (e.g. protein-losing enteropathy)
		- Medications
		- Pelvic compression (e.g. tumor, lymphoma)
		- Renal disease (e.g. nephrotic syndrome)
		- Sodium or fluid overload (e.g. parenteral fluids)
		- Venous insufficiency

Leg edema due to lymphoma is usually bilateral, given its effects on multiple organ systems and progressively impaired fluid return mechanisms. Previous reviews and case reports indicate that lymphoma-related venous drainage issues usually lack B symptoms and that leg edema may be unilateral [6,12]. An abdominal/pelvic CT scan is recommended to rule out tumors when suspicious findings (unilateral edema, pelvic signs, weight loss) are present [5]. In the present case of DLBCL, there were no B symptoms. The patient initially presented with unilateral right-side lower extremity and scrotal edema, which rapidly progressed to bilateral edema over two weeks. This suggests that leg edema caused by lymphoma-associated venous drainage issues can vary from unilateral to bilateral within a relatively short period, depending on the rate and direction of lymphadenopathy development in the pelvis.

The patient presented with unilateral edema over one month, which progressed to bilateral edema two days after the initial visit. It has been reported that edema can be classified as acute or chronic depending on whether it persists for more than 72 hours after onset [5,6]. In cases where chronic unilateral edema rapidly worsens to bilateral edema within a short period, as seen in this case, it is challenging to identify the underlying cause of the edema by simply applying the acute/chronic and unilateral/bilateral classifications (Table 1). We recommend classifying its progression based on intervals of days, weeks, or months instead of simply categorizing it as chronic edema. This approach is expected to improve the accuracy of diagnosing the underlying causes of complex edema cases in future clinical practice.

## Conclusions

This case of DLBCL rapidly progressed from chronic unilateral edema to bilateral edema. In differentiating the cause of edema, it is essential to consider pelvic examination for lymphoma or other rapidly growing tumors, and the clinician should pay close attention to the course and localization of the edema. Among chronic edema cases, some conditions may require follow-up at intervals of a few days or a week. Instead of using the current 72-hour cutoff for classification and grouping as “chronic edema” together, the differential diagnostic approach can be refined by further subdividing the progression of chronic edema into intervals of days, weeks, or longer. It is also essential to educate the patient to report changes in symptoms and imaging findings, and to capture these changes before the next follow-up visit is essential for an accurate evaluation.
